# Serum Leptin Concentration is Associated with Incident Frailty in Older Adults

**DOI:** 10.14336/AD.2016.0819

**Published:** 2017-04-01

**Authors:** Alberto Lana, Ana Valdés-Bécares, Antonio Buño, Fernando Rodríguez-Artalejo, Esther Lopez-Garcia

**Affiliations:** ^1^Department of Medicine, Preventive Medicine and Public Health Area, School of Medicine and Health Sciences, Universidad de Oviedo, Oviedo, Spain; ^2^Department of Preventive Medicine and Public Health, School of Medicine, Universidad Autónoma de Madrid, IdiPaz (Instituto de Investigación Sanitaria Hospital Universitario La Paz), and CIBERESP (CIBER of Epidemiology and Public Health), Madrid, Spain; ^3^Department of Laboratory Medicine, Hospital University La Paz, Madrid, Spain

**Keywords:** older adults, leptin, biological markers, obesity, insulin resistance

## Abstract

Obesity has been associated with higher risk of frailty in older adults, but the pathophysiological mechanisms are unclear. No previous study has examined the association between leptin, an adipokine, and the risk of frailty in older adults, and whether this association could be explained by insulin resistance or chronic inflammation. Data were taken from 1,573 individuals without diabetes mellitus, aged ≥60 years, from the Seniors-ENRICA cohort. In 2008-2010, leptin, the homeostasis model assessment of insulin resistance (HOMA-IR) and C-reactive protein (CRP) were measured. Study participants were followed-up through 2012 to assess incident frailty, defined as at least two of the following Fried criteria: exhaustion, weakness, low physical activity, and slow walking speed. Analyses were performed with logistic regression and adjusted for the main confounders. Over a median follow-up of 3.5 years, 280 cases of incident frailty were identified. Compared to individuals in the lowest tertile of serum leptin, those in the highest tertile showed an increased risk of frailty (odds ratio [OR]: 2.12; 95% confidence interval [CI]: 1.47-3.06; p-trend <0.001). Further adjustment for the percentage of body fat led to an OR of 1.69 (95% CI: 1.11-2.61; p-trend=0.01). After additional adjustment for HOMA-IR and CRP, the OR for frailty was 1.59 (95% CI: 1.01-2.52; p-trend=0.04). Results did not vary according to sex, abdominal obesity or the percentage of body fat. Being in the highest versus lowest tertile of leptin was associated with increased risk of exhaustion (OR: 2.16; 95% CI: 1.32-3.55; p-trend=0.001) and muscle weakness (OR: 1.77; 95% CI: 1.25-2.51; p-trend=0.001), in the analyses adjusted for potential confounders and body fat. Higher leptin concentration was associated with greater risk of frailty in older adults. This association was only modestly explained by insulin resistance and chronic inflammation, as measured by CRP.

Frailty is a common geriatric medical syndrome whose frequency is expected to increase over the next decades due to the progressive ageing of the population. The frailty syndrome is characterized by an augmented risk of falls, hospitalization, disability and death after exposure to even minor stressors, such as a mild infection or a new drug treatment (1-2). Frailty results from a decline in multiple physiological systems, including the endocrine system. Specifically, general and abdominal obesity are known risk factors for frailty (3-6). Also, obesity-related biomarkers of altered glucose metabolism and chronic inflammation have been linked to frailty. For instance, higher insulin resistance (IR) and C-reactive protein (CRP) have been prospectively associated with greater risk of frailty among adults aged 69 to 74 years participating in the Cardiovascular Heart Study (CHS) (7).

Adipokines (proteins secreted by the adipose tissue) are considered to be a link, at least partially, between obesity and its related morbidity and mortality (8). Leptin, the first adipokine discovered, is secreted mainly by white adipocyte tissue, and its serum levels are positively correlated with fat mass; therefore, serum leptin reflects primarily the amount of energy stored in adipose tissue (9,10). It is known that leptin contributes to the regulation of energy balance by acting on multiple central (brain) and peripheral tissues, but its role in health and disease is still incompletely understood. Among healthy individuals, higher serum leptin leads to increased satiation and reduced food intake as well as higher energy expenditure (9,10). However, serum leptin is usually elevated in obese individuals, which suggests that there might be a state of leptin resistance, whereby leptin is not able to control obesity by appropriately reducing food consumption (11). Also there is some evidence that leptin upregulates proinflammatory cytokines such as tumor necrosis factor-alpha and interleukin-6, and that these are associated with IR (9,10). However, to our knowledge, no previous study has investigated the association between leptin and frailty, together with the role of IR and chronic inflammation.

Accordingly, in a cohort of community-dwelling older adults we examined the association between serum leptin concentrations and the risk of frailty, and whether it could be explained by IR and chronic inflammation. Moreover, we assessed whether the study association was modified by fat mass.

## MATERIALS AND METHODS

### Study design and participants

We used data from the Seniors-ENRICA cohort, whose methods have been reported elsewhere (6,12). Briefly, this population-based cohort was established in 2008-2010 with 2,614 individuals aged ≥60 years in Spain. Baseline information was collected using two procedures. The first was a phone interview on health status, lifestyle, morbidity, and health services use. This interview was followed by two consecutive home visits to collect 12-h fasting blood and urine samples, perform a physical exam, and obtain a diet history. In 2012, a second wave of data collection was performed using the same procedures for data collection. During follow-up, 95 individuals died and 115 were lost, so information was obtained for only 2,404 persons in 2012. The socio-demographic, lifestyle and clinical characteristics at baseline were similar in those who provided updated information on frailty at 2012 and those who did not (13).

The Clinical Research Ethics Committee of the *‘La Paz’* University Hospital approved the study protocol. All study participants gave written informed consent.

### Study variables

#### Frailty

We used a modification of the operational definition proposed by Fried et al. in the CHS (1). Specifically, frailty was defined as having at least two of the following four criteria: a) exhaustion, based on reporting “at least 3 to 4 days a week” for any of two statements from the Depression Scale of the Center for Epidemiological Studies: “I felt that anything I did was a big effort” or “I felt that I could not keep on doing things”; b) weakness, defined as the lowest quintile in the CHS of maximum strength on the dominant hand adjusted for sex and body mass index. We selected the highest value of grip strength in two consecutive measures using a Jamar hand held dynamometer; c) low physical activity, defined as walking ≤2.5 h/week in men and ≤2 h/week in women; and d) slow walking speed, defined as the highest quintile in our study sample for the three-meter walking speed test, stratified for sex and BMI. As in previous studies on the association between diet and risk of frailty (14,15), in the main analyses we excluded the “weight loss” criterion from the definition of frailty because overfeeding is a main cause of both leptin elevation and weight gain (9), and thus may confound a potential association between leptin and weight loss. However, in sensitivity analyses we also included the “weight loss” criterion, so that frailty was defined as the presence of at least three of the five Fried criteria, including a weight loss ≥4.5 kg in the preceding year.

#### Leptin and other biomarkers

Baseline serum leptin concentration (ng/mL) was determined by enzyme-linked immunoassay (Diagnosis Biochem Canada) using a BEST2000 robot. The sensitivity of this test was 0.5 ng/dL and the coefficients of variation intra- and inter-assay were 7.5 and 9.6%, respectively. Fasting glucose (mg/dL) was measured by the glucose oxidase method, and fasting insulin (µU/mL) by immunoradiometric assay. The homeostatic model assessment for IR index (HOMA-IR) was calculated by multiplying fasting glucose by fasting insulin and dividing by 405 (16). Finally, high-sensitivity CRP (mg/L) was determined by latex-enhanced nephelometry. The coefficients of variation intra- and inter-assay were <4% and <4% for glucose, 5.2 and 6.9% for insulin and 3.2 and 5.9% for CRP.

#### Other variables

We also collected baseline data on some potential confounders of the association between leptin and frailty. Specifically, we asked study participants about socio-demographic factors and lifestyle behaviors, such as sex, age, education, tobacco and alcohol consumption, leisure-time physical activity (17), and time spent watching TV. Food consumption was ascertained with a validated diet history, developed from that used in the EPIC-cohort in Spain (18). From this, we calculated the total energy intake and the Trichopoulou index (19); a higher score in this index indicates better adherence to the Mediterranean diet. We also collected health status variables. First, waist circumference, weight and height were measured by trained staff under standardized conditions (12). Abdominal obesity was defined as waist circumference >102 cm in men and >88 cm in women. Body mass index (BMI) was calculated as weight in kg divided by squared height in m. The CUN-BAE equation was used to estimate the percentage (%) of body fat, based on sex, age and BMI in each study participant (20). Second, participants also reported if they suffered from any of the following physician-diagnosed diseases: cardiovascular disease (myocardial infarction, stroke or heart failure), hypertension, cancer at any site, chronic lung disease (asthma or chronic bronchitis), osteomuscular disease (osteo-arthritis, arthritis, hip fracture), or depression requiring drug treatment. Third, the Lawton-Brody index was used to ascertain limitations in instrumental activities of daily living (IADL), with the questions on subjects’ ability to prepare meals, do household chores, and take care of personal laundry being excluded in men (21). And fourth, individuals reported their self-rated health, which was classified as optimal (excellent, very good, or good) or suboptimal (fair or poor).

### Statistical analysis

Among the 2,404 study participants, we excluded 5 subjects with BMI <18.5 kg/m^2^ and 374 with diabetes mellitus (fasting serum glucose >126 mg/dL or treatment with oral anti-diabetic drugs or insulin), because cachexia, diabetes or its treatment are strongly associated both with leptin and frailty status (22,23). We additionally excluded 419 who were frail at baseline or lacked data on frailty at baseline or at the end of follow-up. Finally, we excluded 25 individuals without blood determinations and 8 with missing data on potential confounders. Therefore, the analyses were conducted with 1,573 nondiabetic individuals.

The association between leptin concentration and the risk of frailty was estimated by odds ratios (OR) and their 95% confidence interval (CI), obtained from logistic regressions. Given that serum leptin did not follow a normal distribution, we used the log-transformed values. Then, participants were classified into sex-specific tertiles, because leptin levels were much higher in women than men; the lowest tertile of leptin was used as reference in the analyses. We built four logistic models. The first one adjusted only for age (years) and sex. The second model additionally adjusted for educational level (primary, secondary or university), smoking (never, former or current), alcohol consumption (never, former, moderate, or heavy drinker, with the threshold between moderate and heavy drinking established as 20 g/d in men and 10 g/d in women the threshold between moderate and heavy drinking), leisure physical activity (quartiles of METs-h/week), energy intake (quartiles of kcal/day), Mediterranean diet score, hypertension, cardiovascular disease, cancer, chronic obstructive lung disease, osteomuscular disease, and depression. A third model was additionally adjusted for percentage of body fat (sex-specific quartiles), as a proxy for the concentration of adipokines other than leptin. Finally, a fourth model further adjusted for HOMA-IR and CRP (mg/dl), to assess if they could explain the association between leptin and frailty. To investigate the linear dose-response relationship, we modeled leptin tertiles as a continuous variable, and the p for linear trend was estimated.

Several sensitivity analyses were run to assess the robustness of results. First, we repeated the analyses using the weight loss criterion of frailty, so that this syndrome was defined as having three or more of the five Fried criteria. Second, we replicated the analyses using the lowest quintile of grip strength in our sample to define weakness. Thus, in this sensitivity analysis, frailty was defined using our own cut-off points for all criteria. Third, given the strong association between frailty, disability and poor health, analyses were also repeated among individuals with better health status (no limitations in IADL, optimal self-rated health or without diagnosed chronic disease). Lastly, to rule out the possibility that sedentary behavior and poor diet lead to both higher leptin level and frailty, the analyses were replicated among those who watched less TV than the sample median (2 h/day) or had a Trichopoulou index higher than the sample median (5 points).

Because the physiology of leptin in the non-obese status may differ from that in obesity, where a resistance to the effect of leptin has been suggested, we conducted analyses stratified by abdominal obesity and the sex-specific median of body fat percentage. To assess if the study association varied across the strata we built interaction terms as the product of leptin tertiles by the stratification variable, and then used likelihood ratio tests to compare models with and without interaction terms. Lastly, we used the same type of analyses to assess the association between leptin level and each frailty criterion.

We tested if the main results varied with sex by using interaction terms. Since the results were similar in each sex and the interactions did not reach statistical significance, the results are reported for the total study sample. A 2-tailed p value <0.05 was considered as statistically significant. Statistical analyses were performed with Stata, version 12.0 (Stata Corp., College Station).

**Table 1:** Characteristics of study participants according to sex-specific tertiles^a^ of serum leptin concentration. (N=1,573)

	Leptin concentration			*p*-trend
	Tertile 1 (lowest)	Tertile 2	Tertile 3 (highest)	
Participants, n	528	531	514	
Age, y	68.4 (6.6)	68.0 (6.0)	68.9 (6.3)	0.17
Men, %	46.6	46.3	46.9	0.93
University education, %	25.2	21.1	19.5	0.03
Current smoker, %	13.1	12.1	9.0	0.04
Moderate drinker, %	56.3	59.7	59.1	0.34
Leisure-time physical activity, MET-h/wk	24.2 (15.8)	22.6 (14.9)	19.3 (14.3)	<0.001
TV watching, h/d	2.2 (1.3)	2.5 (1.4)	2.8 (1.7)	<0.001
Energy, kcal/d	2,040 (556)	2,051 (561)	2,014 (567)	0.46
Trichopoulou index score	4.7 (1.5)	4.5 (1.5)	4.4 (1.6)	0.02
Waist circumference, cm	89.2 (10.5)	95.8 (9.1)	104.2 (10.6)	<0.001
Body fat, %	33.9 (6.5)	36.8 (6.5)	40.5 (7.2)	<0.001
Morbidity, %				
Cardiovascular disease	4.4	4.9	4.5	0.92
Hypertension	54.0	60.6	73.0	<0.001
Chronic lung disease	4.7	7.9	9.5	0.003
Cancer	1.5	2.3	1.8	0.77
Osteomuscular disease	40.7	49.2	52.3	<0.001
Depression	6.4	7.0	9.5	0.06
Independent in IADL, %	91.3	90.2	88.3	0.02
Optimal self-rated health, %	78.8	71.0	62.8	<0.001
HOMA-IR	1.4 (1.0)	2.0 (1.1)	3.0 (2.0)	<0.001
CRP, mg/L	0.25 (0.4)	0.39 (0.9)	0.43 (0.7)	<0.001

For continuous variables mean and standard deviation (SD) are reported.

MET-Metabolic equivalent; TV: Television; IADL: Instrumental activities of daily living; HOMA-IR: Homeostasis model assessment of insulin resistance; CRP: C-reactive protein.

a Sex-specific tertile cut-points for leptin were 6.5 and 13.6 ng/mL in men and 22.5 and 37.0 ng/mL in women

## RESULTS

Among the study participants, the mean (±standard deviation) serum leptin concentration was 23.3 (±19.8) ng/mL, and it was significantly higher in women (33.1±21.2) than in men (12.1±9.7). Table 1 shows the socio-demographic, behavioral and health status characteristics of the participants according to the sex-specific leptin tertiles. Compared to those in the lowest tertile, those with higher leptin concentrations had lower education, were less frequently current smokers, performed less recreational physical activity, spent longer time watching TV, and presented lower adherence to the Mediterranean diet, with higher waist circumference and higher body fat percentage. Moreover, they showed a higher frequency of hypertension, chronic lung disease or osteomuscular diseases as well as lower frequency of IADL-independence and optimal self-rated health. Finally, higher leptin levels were positively associated with HOMA-IR and CRP concentration.

**Table 2:** Odds ratios (95% confidence interval) for the association between serum concentration of leptin and risk of frailty, in the total study sample and according to characteristics of study participants. (N=1,573)

	Leptin concentration			*p*-trend
	Tertile 1 (lowest)	Tertile 2	Tertile 3 (highest)	
Overall				
Participants, n	528	531	514	
Frailty cases	65	82	133	
Model 1^a^	1.00	1.38 (0.96-1.99)	2.57 (1.83-3.60)	<0.001
Model 2^b^	1.00	1.27 (0.87-1.87)	2.12 (1.47-3.06)	<0.001
Model 3^b^	1.00	1.19 (0.79-1.78)	1.69 (1.11-2.61)	0.01
Model 4^b^	1.00	1.14 (0.75-1.72)	1.59 (1.01-2.52)	0.04
Sensitivity analyses				
Frailty including weight loss				
Participants, n	528	531	514	
Frailty cases	14	23	58	
Model 1^a^	1.00	1.89 (0.94-3.80)	5.26 (2.83-9.80)	<0.001
Model 2^b^	1.00	1.66 (0.80-3.46)	4.25 (2.18-8.29)	<0.001
Model 3^b^	1.00	1.64 (0.76-3.52)	4.09 (1.90-8.79)	<0.001
Model 4^b^	1.00	1.41 (0.65-3.08)	2.95 (1.29-6.72)	0.005
Defining weakness as lowest quintile of grip strength in our sample				
Participants, n	528	531	514	
Frailty cases	55	60	102	
Model 1^a^	1.00	1.15 (0.77-1.72)	2.25 (1.55-3.26)	<0.001
Model 2^b^	1.00	1.04 (0.67-1.59)	1.78 (1.19-1.66)	0.003
Model 3^b^	1.00	1.09 (0.69-1.72)	1.78 (1.10-2.88)	0.01
Model 4^b^	1.00	1.05 (0.66-1.68)	1.67 (1.01-2.80)	0.04
Participants independent in IADL				
Participants, n	478	474	448	
Frailty cases	52	61	104	
Model 1^a^	1.00	1.35 (0.90-2.02)	2.60 (1.79-3.77)	<0.001
Model 2^b^	1.00	1.22 (0.80-1.88)	2.13 (1.43-3.19)	<0.001
Model 3^b^	1.00	1.06 (0.68-1.65)	1.58 (0.99-2.52)	0.04
Model 4^b^	1.00	1.03 (0.65-1.62)	1.50 (0.91-2.49)	0.09
Participants with optimal self-rated health				
Participants, n	44	43	57	
Frailty cases	413	372	320	
Model 1^a^	1.00	1.20 (0.76-1.90)	1.86 (1.20-2.89)	0.006
Model 2^b^	1.00	1.15 (0.71-1.88)	1.66 (1.04-2.65)	0.03
Model 3^b^	1.00	1.03 (0.62-1.71)	1.32 (0.76-2.28)	0.32
Model 4^b^	1.00	1.03 (0.61-1.73)	1.33 (0.73-2.41)	0.35
Participants without diagnosed chronic disease				
Participants, n	284	229	203	
Frailty cases	23	19	31	
Model 1^a^	1.00	1.12 (0.58-2.15)	2.20 (1.22-3.98)	0.009
Model 2^b^	1.00	1.21 (0.61-2.40)	2.17 (1.15-4.12)	0.02
Model 3^b^	1.00	1.05 (0.52-2.12)	1.53 (0.73-3.21)	0.26
Model 4^b^	1.00	1.04(0.51-2.15)	1.51 (0.68-3.35)	0.32
Participants who spent < 2 h/d watching TV				
Participants, n	318	219	235	
Frailty cases	35	31	49	
Model 1^a^	1.00	1.43 (0.87-2.35)	2.23 (1.37-3.63)	0.001
Model 2^b^	1.00	1.30 (0.76-2.24)	1.96 (1.14-3.37)	0.01
Model 3^b^	1.00	1.11 (0.62-1.97)	1.45 (0.77-2.75)	0.24
Model 4^b^	1.00	1.05 (0.58-1.91)	1.33 (0.66-2.66)	0.40
Participants whose diet had a Trichopoulou index ≥ 5				
Participants, n	297	277	245	
Frailty cases	36	44	63	
Model 1^a^	1.00	1.57 (0.96-2.58)	2.61 (1.63-4.20)	<0.001
Model 2^b^	1.00	1.54 (0.90-2.65)	2.36 (1.40-3.98)	0.001
Model 3^b^	1.00	1.27 (0.72-2.26)	1.73 (0.93-3.19)	0.08
Model 4^b^	1.00	1.18 (0.65-2.12)	1.57 (0.81-3.05)	0.18

IADL: Instrumental activities of daily living; TV: Television

a Model 1: logistic regression model adjusted for sex and age (years).

b Model 2: model 1 additionally adjusted for educational level (≤primary, secondary, university), smoking (never, former, current), leisure-time physical activity (quartiles of MET-h/wk), energy intake (quartiles of Kcal/d), alcohol consumption (never, former, moderate drinker, heavy drinker), Trichopoulou index score, hypertension, cardiovascular disease, cancer, chronic lung disease, osteomuscular disease, and depression.

c Model 3: model 2 additionally adjusted for body fat (quartiles of %).

d Model 4: model 3 additionally adjusted for HOMA-IR and CRP (mg/L).

Over a median follow-up of 3.5 years, we identified 280 (17.8%) persons with incident frailty. Using the five-criterion definition of frailty (i.e. including weight loss), there was 95 (6.0%) incident cases. After adjustment for potential confounders except for body fat percentage (model 2), and compared to individuals in the lowest tertile of leptin concentration, the OR (95% CI) for frailty was 1.27 (0.87-1.87) in the second tertile, and 2.12 (2.12-3.06) in the highest tertile (p-trend <0.001). After additional adjustment for body fat percentage, the corresponding results were 1.19 (0.79-1.78) and 1.69 (1.11-2.61; p-trend=0.01). Finally, with further adjustment for HOMA-IR and CRP the results were 1.14 (0.75-1.72) and 1.59 (1.01-2.52; p-trend=0.04) (Table 2). Results were in the same direction when the weight loss criterion was included in the definition of frailty and when sample-specific cut-offs for grip strength were used, as well as among individuals with better health status, and in those with non-sedentary behavior or better adherence to the Mediterranean diet (Table 2).

**Table 3:** Odds ratios (95% confidence interval) for the association between serum concentration of leptin and risk of frailty, by abdominal obesity and body fat (N=1,573).

	Leptin concentration			*p*-trend	*p*-interaction^†^
	Tertile 1 (lowest)	Tertile 2	Tertile 3 (highest)		
Abdominal obesity					
Participants, n	287	288	285		
Frailty cases	46	62	90		
Model 1^a^	1.00	1.15 (0.71-1.86)	1.94 (1.15-3.26)	0.006	
Model 2^b^	1.00	1.09 (0.64-1.85)	1.56 (0.88-2.77)	0.09	
No abdominal obesity					
Participants, n	238	238	237		
Frailty cases	25	19	38		
Model 1^a^	1.00	0.73 (0.38-1.43)	1.74 (0.85-3.56)	0.12	0.12
Model 2^b^	1.00	0.69 (0.33-1.41)	1.72 (0.79-3.72)	0.15	0.16
Body fat % ≥ median in study sample					
Participants, n	262	263	262		
Frailty cases	42	58	86		
Model 1^a^	1.00	1.17 (0.64-2.13)	1.88 (1.08-3.25)	0.001	
Model 2^b^	1.00	1.12 (0.59-2.13)	1.69 (0.93-3.06)	0.02	
Body fat % < median in study sample					
Participants, n	265	260	261		
Frailty cases	25	29	36		
Model 1^a^	1.00	1.37 (0.83-2.28)	2.14 (1.09-4.20)	0.02	0.47
Model 2^b^	1.00	1.38 (0.79-2.40)	2.03 (0.96-4.29)	0.05	0.56

† Comparing the same model in the complementary stratum.

Abdominal obesity: waist circumference >102 cm in men and >88 cm in women

Median of body fat % in study sample: 30.6% in men and 42.1% in women.

a Model 1: logistic regression model adjusted for sex and age (years).

b Model 2: model 1 additionally adjusted for educational level (≤primary, secondary, university), smoking (never, former, current), leisure-time physical activity (quartiles of MET-h/wk), energy intake (quartiles of Kcal/d), alcohol consumption (never, former, moderate drinker, heavy drinker), Trichopoulou index score, hypertension, cardiovascular disease, cancer, chronic lung disease, osteomuscular disease, and depression.

**Table 4:** Odds ratios (95% confidence interval) for the association between plasma concentration of leptin and risk of each frailty criterion. (N=1,573)

	Leptin concentration			*p*-trend
	Tertile 1 (lowest)	Tertile 2	Tertile 3 (highest)	
Participants, n	528	531	514	
Exhaustion				
Criterion present	49	55	96	
Model 1^a^	1.00	1.16 (0.77-1.76)	2.29 (1.57-3.34)	<0.001
Model 2^b^	1.00	1.06 (0.68-1.65)	2.05 (1.36-3.10)	<0.001
Model 3^c^	1.00	1.12 (0.70-1.79)	2.16 (1.32-3.55)	0.001
Model 4^d^	1.00	1.14 (0.71-1.84)	2.24 (1.32-3.80)	0.002
Low physical activity				
Criterion present	67	71	105	
Model 1^a^	1.00	1.06 (0.74-1.52)	1.77 (1.26-2.47)	0.001
Model 2^b^	1.00	0.97 (0.64-1.42)	1.40 (0.97-2.01)	0.06
Model 3^c^	1.00	0.94 (0.63-1.40)	1.18 (0.77-1.81)	0.42
Model 4^d^	1.00	0.91 (0.60-1.37)	1.11 (0.70-1.77)	0.61
Slow walking speed				
Criterion present	64	68	85	
Model 1^a^	1.00	1.10 (0.76-1.15)	1.41 (0.99-2.01)	0.06
Model 2^b^	1.00	1.08 (0.74-1.57)	1.30 (0.90-1.89)	0.16
Model 3^c^	1.00	1.14 (0.77-1.69)	1.48 (0.96-2.29)	0.08
Model 4^d^	1.00	1.01 (0.67-1.52)	1.19 (0.74-1.92)	0.46
Muscle weakness				
Criterion present	132	149	230	
Model 1^a^	1.00	1.26 (0.94-1.69)	2.61 (1.97-3.46)	<0.001
Model 2^b^	1.00	1.17 (0.86-1.58)	2.35 (1.75-3.17)	<0.001
Model 3^c^	1.00	1.05 (0.76-1.43)	1.77 (1.25-2.51)	0.001
Model 4^d^	1.00	1.01 (0.73-1.40)	1.68 (1.15-2.44)	0.005
Weight loss				
Criterion present	31	32	43	
Model 1^a^	1.00	1.04 (0.62-1.73)	1.45 (0.90-2.35)	0.12
Model 2^b^	1.00	1.02 (0.61-1-73)	1.40 (0.84-2.33)	0.18
Model 3^c^	1.00	0.98 (0.57-1.69)	1.34 (0.74-2.42)	0.32
Model 4^d^	1.00	0.97 (0.55-1.67)	1.25 (0.66-2.35)	0.49

a Model 1: logistic regression model adjusted for sex and age (years).

b Model 2: model 1 additionally adjusted for educational level (≤primary, secondary, university), smoking (never, former, current), leisure-time physical activity (quartiles of MET-h/wk), energy intake (quartiles of Kcal/d), alcohol consumption (never, former, moderate drinker, heavy drinker), Trichopoulou index score, hypertension, cardiovascular disease, cancer, chronic lung disease, osteomuscular disease, and depression.

c Model 3: model 2 additionally adjusted for body fat (quartiles of %).

d Model 4: model 3 additionally adjusted for HOMA-IR and CRP (mg/L).

In stratified analyses, the association between leptin and risk of frailty was similar in those with or without abdominal obesity, and in those with body fat percentage above or below the sample median (Table 3).

Table 4 presents the association between leptin concentrations and each frailty criterion. Compared to individuals in the lowest tertile of leptin, those in the highest tertile showed a tendency to increased risk of all criteria, though statistical significance was only achieved for exhaustion (model 3, OR: 2.16; 95% CI: 1.32-3.55; *p*-trend=0.001) and muscle weakness (model 3, OR: 1.77; 95% CI: 1.25-2.51; *p*-trend=0.001).

## DISCUSSION

In this study among community-dwelling older adults, higher leptin serum concentrations were associated with incident frailty. This association was observed regardless of abdominal obesity or body fat percentage, and was only modestly explained by insulin resistance or chronic inflammation, as represented by CRP levels.

To our knowledge, this is the first longitudinal study to examine the risk of frailty associated with leptin concentrations. However, one investigation has found indirect evidence for this association, which is consistent with our results. Specifically, Aguirre et al. have recently reported that the association between high body fat mass and low bone density, which is a predictor of frailty, is mediated by leptin in older adults (24).

In healthy individuals, leptin reduces food intake and increases energy expenditure, so it contributes to energy balance (9,10). However, there are at least two situations that could alter the physiology of leptin and could be related to some deleterious health effects. First, several diseases and severe disability can alter the effect of leptin on the hypothalamus, which is the main target of leptin (9). For instance, low leptin concentrations in patients with cancer are not associated with greater appetite, suggesting a disturbance in the hypothalamic response to leptin (25). Also, another study found low leptin levels in a small sample of institutionalized frail older adults, which was accompanied by increased levels of CRP and interleukin-6 (26). It is possible that, in these cases, low leptin levels simply reflect malnutrition status, which is also related to frailty. To rule out the role of baseline malnutrition in the study association, we excluded from the analyses one individual with BMI <18.5 kg/m^2^. Moreover, the association between leptin and frailty was observed even in those free of IADL limitations, with no diagnosed chronic diseases or with optimal reported health.

Second, given that leptin levels reflect overall adipose mass (9,10), obese people have elevated leptin concentrations, which nevertheless are not able to control obesity by reducing food consumption; in fact, administration of leptin to obese individuals does not lead to weight loss (11). These findings have been interpreted as representing a state of leptin resistance (11). The ageing-associated loss of muscle mass is often accompanied by gains in fat mass, even in the absence of obvious signs of obesity (27). This increase in adipose tissue is mostly located in the abdomen and infiltrating the skeletal muscle. Therefore, the state of hyperleptinemia and leptin resistance that concurs in obese people may similarly affect older individuals, a fact which has already been demonstrated in animals (28). Also, given that both ageing and obesity are risk factors for frailty, an association between higher leptin and increased risk of frailty was to be expected. This is consistent with the association between leptin and muscle weakness observed in our study.

Sarcopenia (reduced skeletal muscle mass and strength) plays a key role in the pathogenesis of frailty, leading to reduced physical activity, lower gait speed and greater fatigue (1,2,29). Among the potential mechanisms is the obesity-derived intracellular lipotoxicity (i.e., elevated intramuscular levels of lipids and their derivatives), which induces apoptosis by means of an elevated oxidative stress (30). Also in obesity, muscle progenitor cells could differentiate to an adipocyte-like phenotype as a result of paracrine signals from adipo-cytokines leading to a reduced muscular renewal capacity (30). Interestingly, there is evidence that leptin concentrations are elevated in sarcopenic obesity (31), which in turn is an important risk factor for frailty (1,2,32). Lastly, in a recent study of 139,691 subjects from 17 countries, reduced grip strength has been prospectively linked to higher risk of cardiovascular and all-cause death (33). Therefore, our results are of particular importance because they showed an association of leptin with both frailty and muscle weakness, each of them being a clinically relevant outcome.

It is known that higher leptin may lead to higher production of proinflammatory cytokines, which are related to IR (9,10). In fact, in our cohort, being in a higher leptin tertile was associated with higher HOMA-IR and CRP. Thus, we hypothesized that the leptin-frailty association could be mediated, at least partially, by IR and inflammation. However, the adjusted OR of frailty for the highest versus lowest tertile of leptin was 1.69 and it was reduced to only 1.59 after additional adjustment for HOMA-IR and CRP. This suggests that the study association was only modestly explained by HOMA-IR and CRP. However, the role of other biomarkers of inflammation or oxidative stress, such as interleukin-6, tumor necrosis factor-alpha or reactive oxygen metabolites, cannot be entirely ruled out because they were not measured in this study.

Lastly, we found no evidence that abdominal obesity or body fat percentage modified the association between leptin and frailty. However, stratified analyses lead to smaller sample sizes and may limit the power to find statistical interactions. Moreover, body fat percentage was estimated with the CUN-BAE equation rather than directly measured. Therefore, our results should be confirmed in future studies with a larger sample size and direct measures of fat mass.

Our study had several strengths and limitations. Among the first, is that most variables, including leptin and frailty, were measured with standardized and valid methods. Also the analyses adjusted for a good number of potential confounders, and the main results held after many sensitivity analyses. The main limitation was the lack of measurement of the soluble receptor of leptin, which has shown a stronger relation than leptin with some health outcomes (34). Neither did we measure other adipokines, particularly adiponectin; it seems that the joint assessment of leptin and adiponectin may be more biologically relevant than of leptin alone (10,35). However, we have attempted to partially account for this limitation by adjusting the analyses for body fat percentage, as a proxy for adipokines secretion.

### Conclusion

In conclusion, in this prospective study of older adults, serum leptin concentrations were positively associated with incident frailty. Future research should confirm this finding. Future studies should also establish whether this association could be better characterized by the soluble receptor of leptin, and whether it is independent of the role of other adipokines. Moreover, the mechanisms of this association need to be elucidated.
